# Viral epidemiology and SARS‐CoV‐2 co‐infections with other respiratory viruses during the first COVID‐19 wave in Paris, France

**DOI:** 10.1111/irv.12853

**Published:** 2021-04-04

**Authors:** Quentin Le Hingrat, Donia Bouzid, Christophe Choquet, Odile Laurent, François‐Xavier Lescure, Jean‐François Timsit, Nadhira Houhou‐Fidouh, Enrique Casalino, Jean‐Christophe Lucet, Diane Descamps, Benoit Visseaux

**Affiliations:** ^1^ INSERM IAME Université de Paris Paris France; ^2^ AP‐HP Nord Virology Department Bichat‐Claude Bernard University Hospital Paris France; ^3^ AP‐HP Nord Emergency Department Bichat‐Claude Bernard University Hospital Paris France; ^4^ AP‐HP Nord Infectious Diseases Department Bichat‐Claude Bernard University Hospital Paris France; ^5^ AP‐HP Nord Medical and Infectious Diseases Intensive Care Unit Bichat‐Claude Bernard University Hospital Paris France; ^6^ AP‐HP Nord Infection Control Unit Bichat‐Claude Bernard University Hospital Paris France

**Keywords:** respiratory viruses, SARS‐CoV‐2, syndromic testing, viral co‐infection

## Abstract

**Objectives:**

Our work assessed the prevalence of co‐infections in patients with SARS‐CoV‐2.

**Methods:**

All patients hospitalized in a Parisian hospital during the first wave of COVID‐19 were tested by multiplex PCR if they presented ILI symptoms.

**Results:**

A total of 806 patients (21%) were positive for SARS‐CoV‐2, 755 (20%) were positive for other respiratory viruses. Among the SARS‐CoV‐2‐positive patients, 49 (6%) had viral co‐infections. They presented similar age, symptoms, except for fever (*P* = .013) and headaches (*P* = .048), than single SARS‐CoV‐2 infections.

**Conclusions:**

SARS‐CoV‐2‐infected patients presenting viral co‐infections had similar clinical characteristics and prognosis than patients solely infected with SARS‐CoV‐2.

## 1 INTRODUCTION

As of December 2020, COVID‐19 has been responsible for more than 63 million infections and over 1.5 million deaths worldwide.^1^ The common symptoms of COVID‐19 are fever, cough, dyspnea, fatigue, myalgia, and diarrhea. Most of these symptoms are also falling within the definition of influenza‐like illness (ILI).^2^, ^3^ In France, SARS‐CoV‐2 first wave struck from late February to the end of April, coinciding with the very end of the winter‐associated viruses' epidemic this year and raising the question of potential viral co‐infections with SARS‐CoV‐2 and their impact.

A few studies reported cases of SARS‐CoV‐2 co‐infection with other respiratory viruses, and a meta‐analysis estimated the prevalence of those viral co‐infections to 3%.^4^, ^5^, ^6^, ^7^ However, none of the included studies has used systematic wide range PCR methods. Moreover, there are no data on the impact of SARS‐CoV‐2 viral co‐infection on disease severity and clinical outcomes.

Here, we take advantage of the systematic multiplex PCR (mPCR) testing of patients hospitalized for respiratory tract infection during the first SARS‐CoV‐2 epidemic wave to assess the cocirculation of all respiratory viruses with SARS‐CoV‐2, the number of SARS‐CoV‐2 viral co‐infections, and the clinical features of such co‐infections.

## 2 METHODS

All adult patients hospitalized in a COVID‐19 first‐line hospital in Paris, France, from January 25, 2020, to April 30, 2020, were included. All patients were tested by systematic mPCR testing if they presented ILI symptoms, according to the eCDC definition, and required hospitalization. The two mPCR assays used during the study period, the QIAstat‐Dx SARS‐CoV‐2 respiratory panel, Qiagen,^8^ and the BioFire FilmArray RP2+, BioMérieux,^9^ allow for detecting a wide range of viral and atypical bacterial respiratory targets, including influenza A and B, parainfluenza virus, rhinoviruses/enteroviruses, RSV, metapneumovirus, adenovirus, human coronaviruses (229E, HKU1, OC43, and NL63), Mycoplasma pneumoniae, and Bordetella pertussis. According to the French national definition, a specific SARS‐CoV‐2 RT‐PCR for at‐risk patients was performed, starting from March 10, 2020, when systematic testing for SARS‐CoV‐2 infection began. During this period, three SARS‐CoV‐2 RT‐PCR assays were used: the WHO‐recommended in‐house RT‐PCR assay, the RealStar® SARS‐CoV‐2 RT‐PCR kit, and the Cobas® SARS‐CoV‐2 assay (Roche Diagnostics). All these assays provided similar performance and limit of detection.^10^, ^11^ Demographic, clinical, and biological features were prospectively collected in the Emergency Department (ED) and retrospectively from the other units. Baseline characteristics within each group were summarized using appropriate descriptive statistics. The statistical analysis was performed using Stata15. The research was approved by the local ethic committee N° CER‐2020‐6 (Table 1).

## 3 RESULTS

**FIGURE 1:**
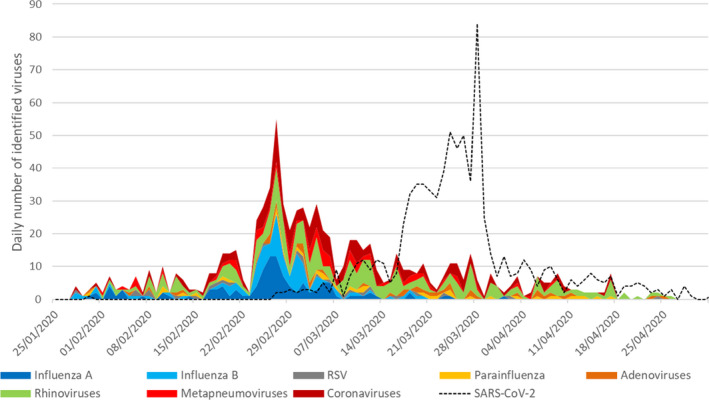
Temporal distribution of SARS‐CoV‐2 and other respiratory viruses during the study period

A total of 3768 patients were included during the study period, 1906 from the ED and 1862 other inpatients. Overall, 806 (21%), 755 (20%), and 28 (1%) samples were positive for SARS‐CoV‐2, any other respiratory viruses, or atypical bacteria, respectively. The SARS‐CoV‐2 wave struck from February to April with a peak incidence between mid‐March and early‐April. At this time, rhinoviruses, human coronaviruses, adenoviruses, or parainfluenza viruses were still circulating, but most of the seasonal respiratory virus epidemic was already gone, especially influenza (cf Figure 1). Among the 806 SARS‐CoV‐2‐positive patients, 42 (5%), 7 (1%), and 6 (1%) also presented one virus, two other viruses, and atypical bacteria, respectively, and 61 (8%) non‐SARS‐CoV‐2 patients positive by mPCR showed viral co‐infections. Most frequently associated viruses were rhinoviruses (17), common human coronaviruses (15), adenoviruses (7), parainfluenza (5), metapneumovirus (4), influenza (4), and RSV (2), and other pathogens (7). The temporal distribution of pathogens is depicted below (Figure 1). When comparing ED patients with SARS‐CoV‐2 either alone (n = 249) or associated (n = 33), they presented similar age, symptoms, vital signs measurements, or comorbidities, except for fever (*P* = .013) and headaches (*P* = .048) (Table 1). Among those co‐infected patients, 0 (0%) were hospitalized in ICU at day 1 versus 64/249 (26%) patients with only a SARS‐CoV‐2 infection, *P* = .78. 5 (2%) among the co‐infected patients died during hospitalization, and 49 (20%) with only a SARS‐CoV‐2 infection, *P* = .78.

**TABLE 1:** Main clinical characteristics of patients presenting a SARS‐CoV‐2 infection with or without any other respiratory virus co‐infection

	SARS‐CoV‐2 only (n = 249)	SARS‐CoV‐2 co‐infection with another respiratory virus (n = 33)	*P*‐value
Age (median, IQR)	59 (49‐73)	58 (45‐69)	.26
Male gender	175 (70%)	24 (73%)	.84
Fever	202 (81%)	21 (64%)	.037
Dyspnea	137 (55%)	16 (48%)	.57
Expectoration	10 (5%)	2 (10%)	.6
Cough	184 (74%)	22 (67%)	.4
Myalgia	74 (30%)	10 (30%)	1
Headache	36 (14%)	10 (30%)	.04
Symptoms duration, median (IQR)	4 [3‐7]	3 [2‐7]	.26
ICU admission, day 1	64 (26%)	0 (0%)	<.001
ICU admission, day 14	31 (15%)	3 (9%)	.27
Hospital mortality	49 (20%)	5 (15%)	.48

## 4 DISCUSSION

This work highlights that 6% of SARS‐CoV‐2‐infected patient presented with viral co‐infection at our adult ED. This proportion is higher than previously reported for SARS‐CoV‐2^6^ but at a level similar to the other respiratory viruses.^12^ This high prevalence of viral co‐infections was observed, despite the limited circulation of other respiratory viruses due to lockdown, curfew, and being in the tail of the season of respiratory viruses.^7^ Rhinoviruses, adenoviruses, and other coronaviruses were the most frequently detected viruses with SARS‐CoV‐2. Adenoviruses and rhinoviruses have already been reported, outside the scope of SARS‐CoV‐2, as being more frequently involved in viral co‐infection, contrary to influenza viruses.^13^

In our population, only 6 patients with SARS‐CoV‐2 were also infected with atypical bacteria. Co‐infections can lead to viral interference, one virus limiting or suppressing the replication of the second virus, or to an enhancement of disease severity compared to mono‐infection.^14^ In our cohort, patients presenting with viral co‐infections with SARS‐CoV‐2 had similar clinical pictures, except for headache and fever, and prognosis than patients solely infected with SARS‐CoV‐2.

Our study presents several strengths and limitations. It showed a relatively large number of SARS‐CoV‐2 co‐infections compared to previous works^4^, ^5^, ^6^, ^7^ and linked virological data with detailed clinical data. Syndromic mPCR testing was performed on all patients presenting with ILI during the study period. Thus, patients recruited in this observational study are not skewed toward more severe patients and represent all adult patients hospitalized for ILI. However, our study is monocentric, and the SARS‐CoV‐2 epidemic flared in Ile‐de‐France when the incidence of most respiratory viruses was waning. Prevalence of viral co‐infections with SARS‐CoV‐2 might be higher in settings with an active circulation of respiratory viruses and/or once social distancing will be over. We also cannot rule out that some specific co‐infections might have a deleterious impact, notably SARS‐CoV‐2/influenza, as only 4 were detected during our study period. Higher severity of SARS‐CoV‐2/influenza A H1N1pdm2009 has recently been described in golden Syrian hamsters when the two viruses were simultaneously inoculated.^15^ We also did not retrieve data on the other pneumonia diagnosis related to *pneumococcus* or *staphylococcus*. Thus, although we found that SARS‐CoV‐2 viral co‐infections were rare during the first epidemic wave and did not differ either by their clinical presentation or by their outcome from SARS‐CoV‐2 mono‐infections, this reassuring finding must be confirmed in the upcoming months.

## CONFLICT OF INTEREST

None.

## DATA AVAILABILITY STATEMENT

The data that support the findings of this study are available from the corresponding author upon reasonable request.

## AUTHOR CONTRIBUTIONS

**Donia Bouzid:** Conceptualization (equal); Formal analysis (equal); Investigation (equal); Methodology (equal); Writing – original draft (equal). **Quentin Le hingrat:** Conceptualization (equal); Investigation (equal); Methodology (equal); Writing – original draft (equal). **Christophe Choquet:** Investigation (equal); Project administration (equal); Writing – original draft (equal). **odile Laurent:** Investigation (equal); Software (equal). **Xavier Lescure:** Supervision (equal); Writing – review and editing (equal). **Jean François Timsit:** Validation (equal); Writing – review and editing (equal). **Nadhira Houhou Fidouh:** Writing – review and editing (equal). **Enrique Casalino:** Supervision (equal); Validation (equal); Writing – review and editing (equal). **Jean Christophe Lucet:** Writing – review and editing (equal). **Diane Descamps:** Validation (equal); Writing – review and editing (equal). **Benoit Visseaux:** Supervision (equal); Validation (equal); Writing – review and editing (equal).

## TRANSPARENCY DECLARATIONS

DB and BV have received funds for speaking at symposia organized on behalf of Qiagen and have also received funds for research from Qiagen.

### PEER REVIEW

The peer review history for this article is available at https://publons.com/publon/10.1111/irv.12853.
